# Getting Started in Tiling Microarray Analysis

**DOI:** 10.1371/journal.pcbi.0030183

**Published:** 2007-10-26

**Authors:** X. Shirley Liu

**Affiliations:** Princeton University, United States of America

## Introduction

The availability of sequenced eukaryotic genomes and commercial oligonucleotide tiling microarrays has enabled many genomics applications. Different from expression microarrays, tiling microarrays have probes that cover the entire genome or contigs of the genome in an unbiased fashion. Currently three commercial sources provide tiling microarrays with different probe lengths and spacing, and array design characteristics. Affymetrix tiles 6 million 25-mer probes per array, which offers the lowest price per probe and the highest resolution (chromosomal distance between neighboring probe centers). Its arrays use one-color assays, so individual samples are hybridized to different arrays. NimbleGen can tile 385,000 50- to 75-mer probes, and Agilent can tile 244,000 60-mer probes per array. The latter two platforms, with longer oligonucleotide probes and two-color assays for which treatment and control samples are differentially labeled and put on the same array for competitive hybridization, have slightly better sensitivity. They are also flexible for custom array design, especially Agilent's multiplex arrays, which allow multiple samples to hybridize on different subareas of the same array. These tiling arrays offer diverse genomic applications, each with its own data analysis challenges.

## ChIP-Chip

The most popular application for the tiling array platform is ChIP-chip, which maps the genome-wide binding locations of transcription factors and other DNA-binding proteins. In a ChIP-chip experiment, chromatin is crosslinked and fragmented to approximately 500 bp. An antibody to the protein of interest is used to precipitate the protein together with its interacting DNA (chromatin immunoprecipitation, or “ChIP”). The coprecipitated DNA is detected on a DNA microarray (the “chip”) and mapped back to the genome [1,2]. In complex genomes, DNA-binding proteins often have thousands of binding sites throughout the genome, so genome tiling microarrays from Affymetrix [3], NimbleGen [4], and Agilent [5] can be used for unbiased binding site mapping.

For ChIP-chip on Affymetrix tiling microarrays, MAT (model-based analysis of tiling arrays) [6] is a very effective peak-finding algorithm. MAT standardizes probe behavior by its 25-mer probe sequence and genome copy number, and can work even without replicate ChIP or control samples. Occasionally Affymetrix genome tiling microarrays have blob-like image defects, which are visible when the array image is converted to a data .cel file. If users encounter array images with blob defects, they are advised to use a “microarray blob remover” [7] to detect and remove affected probes before running MAT. For NimbleGen tiling microarrays, TAMAL [8] is the best algorithm for locating binding sites, while MA2C [9] and TileScope [10] offer alternatives that are more user-friendly and flexible. For Agilent tiling arrays, the joint binding deconvolution [11] algorithm can detect ChIP-chip peaks, in addition providing finer peak spatial resolution than Agilent array tiling resolution.

After the ChIP-chip peaks are detected, biologists often want to find the sequence-specific binding motifs of their protein of interests. MEME [12] and Gibbs Motif Sampler [13] are the most popular tools for de novo motif discovery. As an alternative, biologists could use the cis-regulatory element annotation system [14] to annotate large-scale ChIP-chip data in human and mouse, such as retrieving ChIP-chip sequences, mapping nearby genes, plotting sequence conservation figures, and finding enriched known transcription factor motifs. For a more generalized genomics annotation pipeline, Galaxy (http://main.g2.bx.psu.edu/) offers more customized and interactive features to analyze additional sequenced genomes.

## MeDIP-Chip and DNase-Chip

DNA methylation status often controls gene transcription status, and genome-wide DNA methylation sites can be mapped using methyl–DNA immunoprecipitation followed by microarray (MeDIP-chip). MeDIP-chip is similar to ChIP-chip in protocol, except that an antibody against 5-methyl-cytosine is used to directly precipitate methylated DNA [15,16]. Peak identification and annotation of MeDIP-chip experiments can be conducted with methods similar to ChIP-chip. The methylation level measured by MeDIP-chip should be calibrated by the GC content of the region, since poorly methylated CG-rich regions might still have a higher number of methyl-Cs to MeDIP than fully methylated CG-poor regions.

DNase-hypersensitive regions in the genome are often open chromatin harboring transcriptionally active or regulatory regions, which can be located using DNase-chip. Relying on the assumption that open chromatin is cleaved more often by DNase over a short distance, this experiment involves digesting chromatin with DNase I, isolating DNA fragments created by two DNase cleavages less than 1,200 bp apart, and hybridizing the DNA to tiling microarrays [17]. The resulting tiling array data can be analyzed with a regular ChIP-chip peak-finding algorithm, although window size needs to be adjusted based on the DNA fragment length distribution resulting from the level of DNase digestion.

## Nucleosome Localization

A nucleosome, which consists of ∼146 bp of DNA wrapped around eight histone proteins, forms the fundamental structural unit of eukaryotic chromatin. Since nucleosomes limit DNA accessibility to regulatory factors, it is important to map positioned nucleosomes or nucleosome-free regions in the genome. Nucleosome mapping experiments involve digesting the chromatin with micrococcal nuclease to remove the linker DNA between neighboring nucleosomes, and isolating the remaining nucleosomal DNA to be labeled and hybridized to a tiling microarray. The controls for such experiments are often naked genomic DNA (without chromatin structure) cleaved with hydroxyl radicals or micrococcal nuclease to the same size distribution. Unlike ChIP-chip, the occupancy difference between positioned nucleosomes and linker regions is often less than 10-fold, and positioned nucleosomes occupy only about 100–200 bp of DNA. This requires the tiling microarray to have both high sensitivity and high resolution. Long oligonucleotide microarrays tiled at 5–20 bp resolution are often custom-made to cover selected genomic regions (e.g., promoters or a few megabases on a chromosome) for this application.

In a nucleosome mapping study conducted in yeast Chromosome III [18], a hidden Markov model was applied. The model defines a stretch of probes with low signals as linkers, six to eight probes that span approximately 146 bp with high signals as well-positioned nucleosomes, and more than eight probes with intermediately high signals as delocalized nucleosomes. A Viterbi algorithm is used to infer the most likely partition of probes along the chromosome into the different nucleosomal states. In a similar study conducted in human promoters [19], wavelet transformation was first used to remove noise from the probe signal, which eliminated the high frequency and low coefficient signals. Laplacian Gaussian edge detection was applied to the smoothed probe signal curve to detect peaks and troughs (zero first derivatives) with a reasonable width as positioned nucleosomes and linker regions, respectively.

## ArrayCGH and Copy Number Variation

In an array-based comparative genome hybridization (arrayCGH) experiment, DNA from normal and diseased individuals are differentially hybridized to microarrays to identify copy number variations in the genome that are potential biomarkers or causal genes of disease [20]. Early microarrays used in arrayCGH studies have long (e.g., BAC clones) and/or sparse probes to cover the genome. Recently, tiling microarrays have been used to improve the copy number variation detection sensitivity and resolution [21]. One method proposes a structural change model to use dynamic programming to segment the genome into a number of regions with different copy numbers; within each region the probe signals (thus genome copy number) are similar [22]. However, selecting the number of regions could be difficult for big genomes with complex copy number variations. Hidden Markov model is also a plausible approach to infer the hidden copy number based on observed probe values. One complication that all arrayCGH applications need to reconcile with is that sample impurities (e.g., patient DNA degradation or heterogeneous tumor DNA) sometimes give rise to noisy signals or non-integer copy numbers.

## Transcriptome Mapping

Hybridizing RNA samples to tiling microarrays is gaining popularity for detecting novel transcripts in sequenced genomes. Early studies often called positive probes based on a probe signal cutoff [23], then defined stretches of genomic regions with a significant number of positive probes as transfrags (transcribed fragments). One study on yeast 4-bp resolution tiling arrays adopted a structural change model similar to that used in arrayCGH [24]. In a more recent study profiling multiple *Drosophila* embryogenesis stages on genome tiling microarrays, a Kruskal-Wallis test (a nonparametric analog of one-way ANOVA) was used to detect a stretch of probes giving differential expression among conditions [25]. In addition, the study checked neighboring transfrags with correlated expression in different conditions to find novel 5′, 3′, or internal exons of previously annotated genes. With more transcriptome conditions profiled at better tiling resolution, more advanced algorithms can be developed to refine transfrag borders and detect differential expression, alternative splicing, and antisense transcripts.

## Prospective

All commercial tiling microarray companies strive to put more probes on the array at reduced cost. This trend seems to follow the Moore's Law observed in the semiconductor industry, which dictates that chips double their density at half the cost every 18 months. A few years from now might see tiling microarrays covering the whole mammalian genome at single-base resolution that cost only a few thousand dollars. Tiling arrays will have much wider applications, and researchers might use them for different experiments and informatically select a subset of the probes for analysis. At the same time, high-throughput sequencing technologies such as 454, Illumina Solexa, and ABI SOLiD are making fast progress as well. If enough coverage can be achieved at a cost similar to tiling microarrays, they might give more sensitive and unbiased results. These technologies each entail different challenges and opportunities for computational biologists to develop efficient analysis algorithms. The competition between the different technology companies will inevitably benefit researchers regardless of the winner. Therefore, we look forward to a very exciting decade of genomics advances ahead. 
